# Telehealth Technology Application in Enhancing Continuous Positive Airway Pressure Adherence in Obstructive Sleep Apnea Patients: A Review of Current Evidence

**DOI:** 10.3389/fmed.2022.877765

**Published:** 2022-05-03

**Authors:** Benjamin Ka Seng Thong, Grace Xin Yun Loh, Jia Jan Lim, Christina Jia Liang Lee, Shu Ning Ting, Hong Peng Li, Qing Yun Li

**Affiliations:** ^1^Department of Respiratory and Critical Care Medicine, Ruijin Hospital, Shanghai Jiao Tong University School of Medicine, Shanghai, China; ^2^Shanghai Jiao Tong University School of Medicine, Shanghai, China

**Keywords:** sleep apnea syndromes, eHealth, telemedicine, compliance, CPAP, telemonitoring

## Abstract

Obstructive sleep apnea (OSA) is a common type of sleep-disordered breathing associated with multiple comorbidities. Continuous positive airway pressure (CPAP) is the first choice for moderate-severe OSA but poor compliance brings a great challenge to its effectiveness. Telehealth interventions ease the follow-up process and allow healthcare facilities to provide consistent care. Fifth-generation wireless transmission technology has also greatly rationalized the wide use of telemedicine. Herein, we review the efficacy of the telehealth system in enhancing CPAP adherence. We recommend applying telemonitoring in clinical practice and advocate the development of a biopsychosocial telemedicine model with the integration of several interventions. Big databases and promising artificial intelligent technologies make clinical decision support systems and predictive models based on these databases possible.

## Introduction

Obstructive sleep apnea (OSA) is a common type of sleep-disordered breathing caused by pharyngeal collapse during sleep (1). It is estimated that approximately 1 billion adults aged 30–69 years suffer from OSA worldwide (2). Several studies revealed comorbidities linked with OSA including cardiovascular, cerebrovascular, metabolic diseases, cancer, etc. (3). It is also associated with reduced quality of life (4), excessive daytime somnolence, elevated risk of accidents (5, 6), and sudden cardiac death (7). Since the 1980s, continuous positive airway pressure (CPAP) established as first-line therapy for moderate to severe OSA (8). However, low adherence to CPAP brings a great challenge to clinical practice (9–11). Several strategies are employed to promote adherence and enhance the efficacy of CPAP, i.e., multiple educational interventions, behavioral therapies, CPAP device modifications, etc. (12). During the past two decades, telehealth technology has been applied to the CPAP field, easing the follow-up process and allowing healthcare personnel to deliver more consistent care. Telehealth is defined as the application of telecommunications and digital communication technologies to deliver and facilitate health services (13). Telehealth adopted mobile health, video, audio, digital images, and telemonitoring (TM) to provide clinical and non-clinical services (14). The 2015 guidelines for telemedicine utilization published by the American Academy of Sleep Medicine (AASM) promoted telehealth technology development, and the coronavirus disease (COVID-19) pandemic has expedited internet-based home telemedicine for the diagnosis and treatment of OSA (15). Recent updates from AASM advocated the provision of high-quality sleep care through telehealth interventions and suggested a significant role of telehealth in maintaining the continuity of sleep health (16). Fifth-generation (5G) wireless transmission technology has increased the possibility of wider telemedicine applications. Herein, we aim to review the efficacy of the telehealth system on CPAP adherence and propose the possibility for future developments.

## Factors Associated With Continuous Positive Airway Pressure Adherence

Patient characteristics such as age (17, 18), gender (17, 19), race (20), and smoking status (21) could affect adherence, although these factors were not consistent determinants of CPAP adherence (12). For example, the adherence to CPAP positively correlated with age was reported in a retrospective study (17), but a large cohort study showed a negative correlation with age, particularly in those aged >75 years old, which may be due to the body sickness, sleeping time, or sleep quality of the elderly can affect the adherence of CPAP (18). Smokers caused decreased CPAP adherence compared to non-smokers (21), which is attributed to those smokers being more susceptible to upper airway discomfort, the greater severity of OSA, and as a result, less likely to take advice from healthcare providers (21).

The severity of OSA or the symptoms affects CPAP compliance. Apnea-hypopnea index (AHI), oxygen desaturation index (ODI), and the severity of excessive daytime sleepiness are positively related to adherence (21–23). Further, side effects of CPAP may influence adherence, like mucosal drying, difficult nasal breathing, claustrophobia, etc. (24, 25). Additionally, patients’ psychosocial factors such as the internal locus of control (26), mental health, personality (27–29), and availability of social support (30) can affect adherence. Patients with greater locus control can overcome the side effect to achieve better adherence (26). Also, social support and bed partner is vital in improving CPAP compliance. The sleeping partner could give feedback and advice on the patients’ symptomatic improvements thus may help to improve the adherence (30). Moreover, those who are motivated to resolve their problems tend to have better adherence (27–29).

The higher income and education level is associated with increased adherence. The socio-economic status is positively correlated with health knowledge and accessibility to healthcare services (31). Medical cost, insufficient time, and transport issues concerning patients reduce CPAP adherence. Thus, CPAP adherence is determined by multifactor and should be individualized and closely monitored to address non-compliance and tolerance when prescribed (12, 25).

## Application of Telemonitoring on the Continuous Positive Airway Pressure Adherence

### Effects of Tele-Monitoring on the Obstructive Sleep Apnea Outcome

TM is a subset of telehealth. Real-time feedback reduces clinical care time and ameliorates physiological health account for the positive short-term TM effect significantly (32–36). TM significantly improves psychological health score of Short Form-12 in TM after 6 months (*p* = 0.05) and also shows a higher positive change in score than standard group (SG) which (9.26 ± 2.09 vs. 0.73 ± 1.78, *p* = 0.003) (37, 38). Additionally, TM possessed significant clinical improvements and reduced side effects, and improved the tidal volume (TG = 9.4 mL/kg vs. SG = 8.7 mL/kg, *p* = 0.022) (39) and the blood pressure (systolic blood pressure reduces by 7.4 mmHg and diastolic blood pressure reduces by 4.1 mmHg) (40). It mitigates disease severity shown by improvements on the Epworth Sleepiness Scale (TG ranges from 3.7 to 4.58, SG ranges from 6.05 to 6.1) (38, 40, 41) and reduces residual AHI (TG = 1.3 ± 1.0 vs. SG = 3.2 ± 3.8, *p* = 0.04) (36). With a combination of patient engagement tools, TM even reduces treatment termination by 2.8–5.6% and reduces mask leakage [(TG = 16.9 L/min vs. SG = 19.4 L/min, *p* < 0.0001) and (TG = 2.7 ± 4.0 L/min vs. SG = 4.1 ± 5.3 L/min, *p* < 0.001)] in two respective studies (32, 33, 42).

TM system decreases patients’ burden of visits by enabling flexible timing and reduced traveling times greatly (36). It also reduces costs by €47.32–153.34 compared to inpatient care (43, 44). However, the greater costs of the advanced device and no reimbursement for telemedicine services from most insurance companies are still barriers to patients of lower socioeconomic status (45). Thus, insurance coverage for telemedicine and subsidizing CPAP devices might solve the patients’ financial concerns and secure long-term benefits. TM able to reduce nursing time but there is increased workload in CPAP technicians (36, 44, 46). Since OSA is associated with multiple comorbidities, we notice a distinct lack of studies exploring the associations between TM effects on those with disability.

### Effects of the Telemonitoring in Enhancing Continuous Positive Airway Pressure Adherence

TM system transmits patients’ data to healthcare providers (HPs), enabling HPs to track CPAP usage and adherence at specific time intervals, i.e., 1, 3, and 6 months and more (Table 1) (35, 38, 40, 47–51). Optimally, the HPs will contact the patients through phone calls, messages, email, or by using the automatic feedback system to reinforce knowledge or encourage patients (40, 47, 48). The controlled pilot study and retrospective study demonstrate TM significantly enhanced CPAP mean daily usage hours and median CPAP usage after 1 month (36, 50), although a randomized controlled trial (RCT) finds no significant effect of TM in this 1 month (49).

**TABLE 1:** Summary of telemonitoring studies.

References	Country	Study design (follow-up), *N* (men)	Mean age ± SD (Yrs)	Intervention vs. comparison (system/device)	Adherence criteria	Major findings
Murase et al. (47)	Japan	RCT (6M), *N* = 483 (407)	TM-group: 60 ± 11 Yrs 3M-group: 60 ± 13 Yrs 1M-group: 61 ± 12 Yrs	(TM-group, *N* = 161): Follow-up every 3 months + monthly telemedicine 3 months-group (3M-group, *N* = 166): Follow-up every 3 months 1 month-group (1M-group, *N* = 156): monthly follow-up Device: S9 or AirSense 10; Resmed Corp or REMstar Auto System One or DreamStation; Philips Corp.	% days with ≥4 h/night of CPAP use ≥70%	CPAP adherence: TM-group: 76.6–79.5%, *p* < 0.01 1M-group: 76.2–78.4%, *p* = 0.03 3M-group: 75.6–74.4%, *p* = 0.24
Rattray et al. (46)	Indianapolis	Prospective, mixed-methods (3M), *N* = 90 (84)	IG: 54.9 ± 13.9 Yrs CG: 56.2 ± 15.5 Yrs	IG (*N* = 38): Telesleep quality improvement program (with AirView, ResMed) CG (*N* = 52): Usual care Device: AirSense-10; ResMed	≥ 4 h/night for >70% of nights	PAPadherence: IG vs. CG: 32 vs. 23%, *p* = 0.470
Pepin et al. (38)	French	RCT (6M), *N* = 306 (226)	Median age: Total: 61.3 Yrs IG: 60.8 Yrs CG: 61.8 Yrs	IG (*N* = 157): CPAP initiation educational program + multimodal telemonitoring CG (*N* = 149): CPAP initiation educational program	Not reported	CPAP compliance: IG vs. CG: 5.28 vs. 4.75 h, *p* = 0.05
Mansell et al. (39)	United Kingdom	Longitudinal within-group repeated measures, *N* = 52 (21)	Total = 62 Yrs	All participants were monitored *via* the modem technology and Encore Anywhere (Philips Respironics) system	% days used >4 h	Increased patient compliance from 90 to 96% (*p* = 0.007), and a change in tidal volumes (9.4 vs. 8.7 mL/kg/ideal body weight, *p* = 0.022).
Turino et al. (44)	Lleida, Spain	Prospective randomized controlled study (3M), *N* = 100 (77)	IG: 56 ± 13 Yrs CG: 54 ± 12 Yrs	IG (*N* = 52): Standard care + CPAP equipped with mobile 2G (GSM/GPRS) technology capable CG (*N* = 48): Instruction session + follow-up Device: AirSense 10; ResMed, Martinsried, Germany	Use of CPAP for ≥4 h/day	Compliance: (*p* = 0.627) CG vs. IG: 4.9 vs. 5.1 h/night
Malhotra et al. (33)	United States	Retrospective Study (90 days), *N* = 128,037	IG: 51.8 ± 13.0 Yrs CG: 52.2 ± 13.4 Yrs	IG (*N* = 42679): Receive myAir (provide real-time feedback and coaching to patients based on their data within AirView) CG (*N* = 85358): Did not use the patient engagement tool	≥4 h/night on at least 70% of nights	IG (87.3%) achieving adherence criteria while CG (70.4%), *p* < 0.001 Average therapy usage was 5.9 (IG) vs. 4.9 h/night in the matched CG, *p* < 0.001
Hwang et al. (48)	Southern California	RT (3M), *N* = 556 (325)	Total: 50.5 ± 12.1 Yrs Usual care: 51.9 ± 13.1 Yrs Tel-Ed: 50.3 ± 11.8 Yrs Tel-TM: 48.8 ± 11.8 Yrs Tel-Both: 50.7 ± 11.7 Yrs	Usual care (*N* = 129): Usual care alone Tel-Ed (*N* = 164): Usual care + telemedicine web-based education Tel-TM (*N* = 125): Usual care + CPAP telemonitoring with automated feedback messaging based on usage data for 90 days Tel-Both (*N* = 138): Usual care + both telemedicine-based education and telemonitoring with feedback messaging Device: AirSense 10; ResMed Corp	≥4 h/night on ≥70% of days	Medicare adherence: Usual care vs. Tel-TM 53.5 vs. 65.5% (Odds ratio 1.7, *p* = 0.003) Usual care vs. Tel-Both: 53.5 vs. 73.2% (Odds ratio 2.4, *p* = 0.001) Average daily used: Usual care vs. Tel-TM: 3.8 vs. 4.4 h, *p* < 0.001 Usual care vs. Tel-Both = 3.8 vs. 4.8 h, *p* < 0.001
Anttalainen et al. (36)	Finland	Retrospective Study (1 year), *N* = 111 (IG: 72%, CG: 70.5%)	IG: 53.9 ± 12.2 Yrs CG: 56.4 ± 11.8 Yrs	IG (*N* = 50): CPAP with Restraxx TM online system CG (*N* = 61): CPAP Device: S9 Elite (ResMed, Sydney, Australia), ResTraxx Online (ResMed, Sydney, Australia) database	>4 h per day	Mean CPAP adherence (IG vs. CG: 6.4 vs. 6.1 h; *p* = 0.63) was good in both groups at 1-year follow-up.
Kotzian et al. (51)	Vienna, Austria	RCT (3M, 12M), *N* = 33 (23)	IG: 62.9 ± 5.3 Yrs CG: 61.8 ± 5.3 Yrs	IG (*N* = 17): Standard care + data monitoring CG (*N* = 16): Standard care Device: AirSense 10 AutoSet CPAP (Resmed)	≥4 h/night	Mean adherence to PAP uses all days: (3M) CG vs. IG: 299 vs. 375 min per day, *p* = 0.017 (12M) CG vs. IG: 307 vs. 352 min per day, *p* = 0.204
Fernandes et al. (49)	Lisbon, Portugal	RCT (4 weeks), *N* = 51 (42)	Total: 54.0 ± 12.6 Yrs IG1: 56.3 ± 12.1 Yrs IG2: 53.5 ± 11.7 Yrs CG: 52.3 ± 15.2 Yrs	IG1 (Phone-call care, *N* = 18): Received regular phone calls (to assess self-reported adherence and address any issues regarding the patient’s clinical status or treatment) IG2 (Telemonitored clinical care, *N* = 12): All CPAP devices were fitted with a ResTraxx wireless transmitter (ResMed) to allow remote data collection of adherence or data were collected and transmitted to the computer server CG (Usual clinical care, *N* = 21): Only attended the follow-up for clinical assessment and data collection Device: AutoSet Spirit S8 flow generator unit (ResMed, San Diego, CA)	≥4 h/night for >70% of nights	Mean: Adherence: IG1 vs. IG2 vs. CG: 3.9 vs. 5.0 vs. 5.1 h/day, *p* > 0.05
Fields et al. (37)	United States	Prospective, parallel-group randomized pilot study (3M), *N* = 34 (32)	Total = 53.2 ± 14.8 Yrs IG = 46.7 ± 13.1 Yrs CG = 58.2 ± 14.4 Yrs	CG (*N* = 20): In-person instruction from experienced sleep therapists IG (*N* = 14): No in-person set-up instruction, according to the instructional DVD and brochure Device: Type 3 portable monitor (Embletta Gold; Embla, Inc., Broomfield, CO) (Automatic Positive Airway Pressure)	Mean daily minutes of PAP use over 3M	The mean days of usage: CG vs. IG: 54 vs. 65 days
Hoet et al. (35)	Brussels, Belgium	RT (3M), *N* = 46 (IG: 17%, CG: 57%)	IG: 59 ± 13 Yrs CG: 54 ± 14 Yrs	CG (*N* = 23): Received written instructions and were able to contact the sleep unit (with a telephone call or visit) as often as needed IG (*N* = 23): Usual care + the T4P (SRETT medical, France) TM unit was added to the CPAP that allows practitioners to obtain data *via* the transmission Device: S9 or Airsense 10 from Resmed or DreamStation from Philips	≥4 h per night on ≥70% of nights	Mean duration of use at 3M: (*p* = 0.018) IG vs. CG: 5.7 vs. 4.2 h/night Mean of the total number of hours used: (*p* = 0.034) IG vs. CG: 507 vs. 387 h Compliance between 6 weeks and 3M: IG, *p* = 0.003 CG, *p* = 0.03
Schoch et al. (52)	Eastern Switzerland	RCT (6M), *N* = 169 (27)	Median age: IG: 55 Yrs CG: 57 Yrs	IG (*N* = 82): Instruction session + CPAP machine (was coupled to a telemetry device that regularly transmitted the acquired data to a secure online depository) + follow-up (at 1 and 6M after CPAP initiation) CG (*N* = 87): Instruction session + CPAP machine + follow-up (at 1 and 6M after CPAP initiation) Device: Automated CPAP devices (ICON + AUTO; Fisher & Paykel)	≥4 h/night	Percentage of nights with CPAP use: IG vs. CG: 92 vs. 88.2%, *p* = 0.565 Average nightly use: IG vs. CG: 5.6 vs. 4.8 h, *p* = 0.663
Nilius et al. (40)	Germany	RT (6M), *N* = 75 (55)	IG: 58.6 ± 9.3 Yrs CG: 55.4 ± 10.4 Yrs	IG (*N* = 38): Based on telemonitoring, telephone calls, and remote interventions CG (*N* = 37): Standard practice Device: Positive pressure device (ICON, Fisher and Paykel healthcare, New Zealand)	>4 h/night	Daily usage: CG vs. IG: 2.1 vs. 4.4 h/night, *p* < 0.001 Days used > 4 h: CG vs. IG: 27.5 vs. 57.3%, *p* < 0.001
Frasnelli et al. (50)	Eastern Switzerland	Controlled pilot study (1M), *N* = 223 (IG: 76%, CG: 78%)	IG: 55 Yrs CG: 55 Yrs	IG (*N* = 113): Telemetry-triggered interventions (S9 wireless module, ResTraxx) CG (*N* = 110): Home therapy (S9 AutoSet/AutoSet Spirit II/Somnobalance/RemStar Auto Aflex)	≥4 h per night	The median CPAP use: *p* < 0.05 IG vs. CG: 5.3 vs. 4.6 h/night The median days of usage: *p* = 0.023 IG vs. CG: 28 vs. 27 days
Woehrle et al. (42)	Germany	Randomized, controlled clinical trials (1 year), *N* = 6,802 (5,070)	IG: 59 ± 13 Yrs CG: 59 ± 13 Yrs	IG (*N* = 3,401): PAP + AirView (a cloud-based remote monitoring system) CG (*N* = 3,401): PAP + healthcare provider, sleep laboratory and/or treating physician	Not reported	At 1-year, the overall therapy termination rate was significantly lower (5.4 vs. 11.0%; *p* < 0.001), and time to therapy termination was significantly longer (348 vs. 337 days; *p* < 0.05) in the IG
Woehrle et al. (32)	Germany	Retrospective study (180 days), *N* = 1,000 (880)	IG: 56 ± 13 Yrs CG: 55 ± 12 Yrs	IG (*N* = 500): Telemonitoring and patient engagement tool (AirView + myAir) CG (*N* = 500): telemonitoring alone (AirView; proactive care)	Termination rate	Therapy termination occurred less often in the IG (*p* < 0.001).

*SD, standard deviation; Yrs, years; M, month; IG, intervention group; CG, comparison group; TM, telemedicine; RT, randomized trial; RCT, randomized controlled trial.*

Randomized trials illustrate CPAP adherence improves after 3-month of TM (35, 48). A shorter time to the first technical intervention might be associated with better compliance (35). Researchers have further confirmed that the combination of TM and education is more effective than TM alone (48). Notably, some prospective studies did not find significant effects at 3 months follow up, which may be attributed to the small sample sizes (37, 44, 46).

TM improves CPAP adherence at 6-month follow-up (38, 40, 47). TM significantly improves CPAP compliance [telemonitor group (TG) = 57.3 ± 34.5% vs. standard group (SG) = 27.5 ± 32.5%, *p* = 0.00025] and daily CPAP usage time (TG = 4.4 ± 2.5 h/night vs. SG = 2.1 ± 2.2 h/night, *p* = 0.000063) (40). A RCT observed a positive effect of multimodal TM in CPAP compliance compared to the usual care group (TG = 5.28 ± 2.23 h vs. SG = 4.75 ± 2.50 h, *p* = 0.05) (38). TM increases the percentage of days with good adherence from 76.6 ± 24.2% to 79.5 ± 22.0% (*p* < 0.01) after 6 months compared to baseline (47). Contrastingly, a RCT reported that TM did not improve overall adherence after 6 months, which might be due to the “ceiling effect” (52).

Finally, the current evidence has not confirmed the long-term effect on CPAP adherence at 1-year follow-up (36, 51). High dropout rates and telemonitoring during the habituation stage may account for the insignificant results (51). Compared to the titration stage, TM during the habituation stage might be another reason that leads to insignificant results (36). Although there are no significant improvements, TM still possesses a non-inferiority therapeutic effect compared to usual care after 1 year. We postulate that personality traits are a vital factor determining the length of effective TM intervention based on other researches (28) and suggest future research on interventions to secure long-term telemonitoring effects.

It should be noticed that most of the recent findings show equivocal effects similar to a meta-analysis which demonstrates a significantly higher mean difference of 0.79 h of CPAP compliance in the TM group for short-term follow-up (<3 months), but not for long-term follow up (45). Although the long-term effects remain contentious, we still recommend including telemonitoring in current clinical practice due to its multiple benefits and non-inferiority compared to usual care.

### Various Telemonitoring Approaches Showed Different Effects for Continuous Positive Airway Pressure Adherence

Several TM approaches are used to improve adherence to CPAP (Table 2).

**TABLE 2:** Summary of telehealth approaches.

References	Country	Study design (follow-up), *N* (men)	Mean age ± SD (Yrs)	Intervention and comparison	Sleep assessment	Adherence criteria	Major findings
**Telephone**							
Willard-Grace et al. (53)	United States	RT (30 days), *N* = 131 (88)	Total: 49.1 ± 12.1 Yrs IG: 48.5 ± 11.6 Yrs CG: 49.5 ± 12.5 Yrs	IG: Health coaching group (*N* = 56) The health coach will receive a list of patients in the health coaching group and contact them by telephone during the study. CG: Usual care group (*N* = 76)	Not reported	≥4 h/night	The proportion using CPAP device at any time in the past 30 days between IG and CG (%): 55.4 vs. 41.3%, *p* = 0.03. The number of hours used on average over the past 30 days between IG and CG: 2.1 ± 2.8 h vs. 1.8 ± 2.7 h, *p* = 0.04.
Pengo et al. (54)	United Kingdom	Prospective randomized study, (6 weeks), *N* = 112 (84)	IG: Positively framed message group: 46.7 ± 12.2 Yrs Negatively framed message group: 47.1 ± 11.7 Yrs CG: 53.5 ± 12.5 Yrs	IG: Positively framed message group (*N* = 26) Method: The patient receives the positively framed message during phone calls. Negatively framed message group (*N* = 37) Method: The patient receives the negatively framed message during phone calls. CG: Standard care group (*N* = 39)	(i) have both a 4% ODI ≥ 5 events/hour and typical symptoms of OSA (Epworth Sleepiness Scale) > 10 points. (ii) 4% ODI > 15 events/hour	> 4 h/night	The CPAP total hours used among IG (positively framed message group and negatively framed message group and CG (mean ± SD): (i) At week-2, 53.7 ± 31.4 h, 35.6 ± 27.4 h, and 40.8 ± 33.5 h, respectively, *p* < 0.05. (ii) At week-6, there was no significant difference among the 3 groups (*p* = 0.679)
**Text message**							
Munafo et al. (56)	United States	Randomized, prospective, non-blinded study (90 days), *N* = 138 (95)	IG: 52.3 ± 10.6 Yrs CG: 50.0 ± 11.7 Yrs	IG: Telehealth group (*N* = 69): The patients will log in to the U-sleep website and if one of the following intervention points had been triggered, the patient will receive notifications *via* automated text/email: (i) No CPAP data for 2 consecutive days (ii) CPAP usage <4 h for 3 consecutive nights (iii) CPAP usage met Medicare criteria for adherence CG: Standard of care group (*N* = 69):	Not reported	≥4 h/night	Thedaily usage of CPAP between IG and CG (mean ± SD): 5.1 ± 1.9 vs. 4.7 ± 2.1, *p* = 0.24. The percentage of the amount of patients’ days CPAP used for more than 4 h between IG and CG (mean ± SD): 70.2 ± 26.7 vs. 63.3 ± 28.5, *p* = 0.17. The number of minutes coaching required per patient between IG and CG (mean ± SD): 23.9 ± 26.3 vs. 58.3 ± 25.0, *p* < 0.001.
Kataria et al. (55)	United States	RCT (30 days), *N* = 19 (not mentioned)	Not reported	IG: Reminder group: The patient received education at the first visit and a nightly text message as a reminder. CG: Standard-of-care group	Not reported	≥4 h/night.	Themean overall PAP compliance percentage between IG and CG (%): (i) At first 7 days (%): 83.9% vs. 55.4%, *p* = 0.04. (ii) At 30 days (%): 58.9% vs. 36.9%, *p* = 0.22.
**APPs**							
Hostler et al. (57)	United States	CCT (11 weeks), *N* = 61	IG: 44.5 ± 11.3 Yrs CG: 42.1 ± 6.8 Yrs	IG: SleepMapper group (*N* = 30) Method: The patient receives a standard education + follow-up + SleepMapper application. CG: Standard care group (*N* = 31)	AHI ≥ 5.0 events/hour	>4 h/night for at least 70% of nights	Thepercentage of any CPAP usage at night between IG and CG (mean ± SD): 78.0 ± 22.0% vs. 55.5 ± 24.0%, *p* < 0.001. The percentage usage of CPAP which > 4 h between IG and CG (mean ± SD): 54.0 ± 27.0% vs. 37.0 ± 25.0%, *p* = 0.02.
Isetta et al. (82)	Spain	RT (6 weeks), *N* = 60 (47)	Total: 56 ± 10 Yrs IG: 56 ± 9 Yrs CG: 54 ± 12 Yrs	APPnea IG: Regular users (*N* = 38) (APPnea use > 66% of all days) Method: (i) Every day, APPnea will send a message to ask the patient to answer three yes/no questions about OSA treatment. (ii) Once a week, patients will be required to provide their body weight. (iii) APPnea possesses the recommendation section about CPAP use and a good lifestyle. (iv) Global summaries of the questionnaire answers are available to the patient in graphical format weekly. CG: Non-regular users (*N* = 22) (APPnea use <66% of all days)	Not reported	Not reported	The mean hours of CPAP use between IG and CG (mean ± SD): 5.5 ± 1.6 h/day vs. 5.0 ± 1.5 h/day. The regular use of “APPnea” can improve CPAP usage.
Baltaxe et al. (59)	Spain	RCT (3 months), *N* = 67 (38)	IG: 68 ± 15.8 Yrs CG: 65 ± 14.7 Yrs	IG: MyPathway group (*N* = 33) The patient receives face-to-face motivational intervention + follow-up through the MyPathway app. CG: Usual care group (*N* = 34)	Not reported	Not reported	No significant difference in the number of hours used per day (*p* = 0.28). However, the patients showed a high acceptance of the “MyPathway” app (mean score of 7.5/10 on the questionnaire) and agreed that it was easy to use (mean score of 8.5/10 on the questionnaire)

*SD, standard deviation; Yrs, years; IG, intervention group; CG, comparison group; CCT, controlled clinical trial; RT, randomized trial; RCT, randomized controlled trial.*

Telephone: Health coaching through the phone improves CPAP usage in the past 30 days (*p* = 0.03) (53). The researchers call the patients in the intervention group three times in 30 days to identify and assist them in immediate problem-solving (53). The HPs checked CPAP usage, understood the obstacles, and provide patients with different solutions. A designed list of responses used to communicate helps solve issues more effectively in contrast to a study showing the negative result (49, 53). These well-designed interventions might account for the positive effect. In addition, other studies determine that positively framed message increases CPAP total use hours in week-2 but the effect diminishes in week-6 (54).

Text message: Text messages for reminding about CPAP use significantly improve overall compliance in the first 7 days, but the effect fades at 30 days (55). Other research utilizing email and automated message methods shows no significant effect on CPAP adherence (56). The different content of responses from HPs in these studies might account for the differing results. The faded effect might be due to reduced motivation, which could possibly be addressed by a lack of interesting content, videos, or further information on CPAP therapy (54).

Apps: During the past 5 years, a total of three new apps (“SleepMapper”; “MyPathway”; “Appnea”) have been reported in detail (57–59). The apps allow HPs to provide real-time feedback and education, it also allowed patients to self-monitor and receive a personalized prescription for CPAP usage. “SleepMapper” significantly increases any CPAP usage at night (SG = 55.5 ± 24.0% vs. “SleepMapper” group = 78.0 ± 22.0%, *p* < 0.001) and usage of CPAP > 4 h (SG = 37.0 ± 25.0% vs. “SleepMapper” group = 54.0 ± 27.0%, *p* = 0.02) compared to the standard care group (57). Compared to new CPAP patients, “Appnea” significantly improves CPAP adherence who frequently used when compared to rarely used patients (5.6 ± 1.4 vs. 4.3 ± 1.3 h/night, *p* = 0.008) (58). In contrast, although participants were satisfied with the “MyPathway” app, there was no significant modification in PAP adherence. Almost a quarter of patients were 70–79 years and senior patients are perhaps less motivated in learning and utilizing new technology, thus resulting in negative outcomes (59). Limitations of current studies are the small sample sizes and the short study duration (57, 58).

Despite inconclusive results, mobile apps, phone calls, and text messages still possess some advantages. Apps remain the most promising approach. Future app development should continue to focus on user-friendly, online educational programs, troubleshooting models, and real-time access to CPAP usage and AHI data for self-monitoring. They can encourage patients to maintain a healthy lifestyle and adhere to given therapy with minimal effort from HPs (60). The required smartphones or tablet devices are usually affordable (61). For telephone calls and text messages which displayed short-term effects, additional modulations are required to enhance adherence. We postulate that a convenient troubleshooting model and active approach are vital in determining the success of the intervention. Actively assisting users to troubleshoot problems shows better results than passively waiting for calls (59). Maintaining long-term compliance with CPAP therapy is challenging. Adherence to CPAP may decline over time exacerbated by the negative attitudes toward the treatment and insufficient support from family members and the healthcare team (62).

### Application of Tele-Education in Improving Continuous Positive Airway Pressure Adherence

Tele-education is the application of technologies to provide distance learning. Patients’ negative perceptions of the benefit and health value of CPAP are the common causes of poor CPAP adherence (63, 64). Patients’ lack of confidence in the therapeutic effect of CPAP results in poor adherence (65, 66). Therefore, education is vital in enhancing patients’ perception of CPAP therapy. Tele-education is a well-known alternative model to educate patients. The modes of tele-education include slide shows, videos, audio, and web-based learning (67). Therefore, patients can watch the educational material as soon as they are available and shorten clinical care time (36). The recent COVID-19 pandemic has also accelerated the development of tele-education.

A few studies have investigated the effect of tele-education (Table 3). RCTs found educational videos have no significant improvement in CPAP adherence (48, 68). Although patients understood the given slideshow and educational booklet, there was no significant improvement in mean hours of CPAP (69). The Health Belief Model states that a change in health behavior is primarily due to health perception instead of knowledge (66). Confidence in the therapeutic effect is a key factor that alters health practice (66). Therefore, non-continuous tele-education alone is insufficient (48, 68, 69). Most studies had a small sample size (68, 69) and a short period of intervention (48, 68, 69), which may also influence the outcome. No examination of patients’ knowledge, attitude, and practice pre-and post-study is also a limitation of existing educational studies.

**TABLE 3:** Summary of tele-education studies.

References	Country	Study design (follow-up), *N* (men)	Mean age ± SD (Yrs)	Intervention vs. comparison	Length of CPAP use	Outcomes
Bakker et al. (71)	United States	RCT (6M), *N* = 83 (55)	Total: 63.9 ± 7.4 Yrs IG: 63.8 ± 8.3 Yrs CG: 63.9 ± 7.4 Yrs	IG: CPAP + standardized motivational enhancement delivered by a psychologist during two appointments and six phone calls over 32 weeks (*N* = 41) CG: CPAP (*N* = 42) Duration: 32 weeks	Not reported	The brief motivational enhancement significantly increased the average nightly use of CPAP time by 99 min more than the CPAP-only group (*p* = 0.003) after 6 months. (IG: 4.4 h/night vs. CG: 3.3 h/night)
Guralnick et al. (68)	United States	RCT (30 days), *N* = 212 (95)	IG: 54.1 Yrs CG: 50.3 Yrs	IG: Watched video which included information to increase knowledge about the consequences of untreated severe OSA and the importance of CPAP adherence + usual care (*N* = 99) CG: Usual care (*N* = 113) Length of video: 4 min and available online	Not reported	No differences in CPAP adherence at 30 days (3.3, 95% Confidence interval 2.8–3.8 h/day video education; vs. 3.5, 95% Confidence interval 3.1 to 4.0 h/day usual care; *p* = 0.44) or during the 30 days after the sleep clinic visit. (IG: 3.3 h/night vs. CG: 3.5 h/night)
Hwang et al. (48)	United States	RCT (3M), *N* = 734 (349)	Total: 49.1 ± 12.5 Yrs IG: 49.1 ± 12.2 Yrs CG: 50.2 ± 12.7 Yrs	IG: Two educational programs (1) Watch a video about the pathophysiology of OSA and the information about CPAP before the CPAP therapy. Duration of education session: 15 min. (2) Email about the instructions to use CPAP during the first week of intervention. (*N* = 380) CG: CPAP (*N* = 354)	>4 h/night for at least 70% of nights	Telemedicine-based education did not significantly improve CPAP adherence but did increase clinic attendance for OSA evaluation. (*p* > 0.05) (average usage on all days, IG: 5.1 ± 2.5 h/night vs. CG: 4.6 ± 2.5 h/night)
Dharmakulaseelan et al. (69)	Canada	Randomized Feasibility Study (6M), *N* = 48 (30)	IG: 71 Yrs CG: 66.0 Yrs	IG: Educational pamphlet and slideshow. Content of education: risk factors, symptoms, consequences, and treatment of poststroke/transient ischemic attack OSA, good sleep hygiene practices + usual care (*N* = 25) CG: Usual care (*N* = 23) Length of slideshow: 5 min	>4 h/night for at least 70% of nights or ≥28 h/week	No significant difference in mean hours of CPAP use at the 6M follow-up. (IG: 36.4 h/week, vs. CG: 41.9 h/week)

*M, months; SD, standard deviation; Yrs, years; IG, intervention group; CG, comparison group; RCT, randomized controlled trial.*

Educational intervention with motivation enhancement significantly improves CPAP adherence (70). Motivation enhancement is a behavioral intervention based on the principle of the motivational interview (71). Patients had a one-on-one conversation session with a psychologist, watched an educational video, and received follow-up phone calls from the psychologist (71). This intervention significantly increased the average nightly use of CPAP time by 99 min more than the CPAP-only group (*p* = 0.003) after 6 months (71).

The development of tele-technology concerning OSA patients’ psychology and social life remains scarce. Multiple healthcare-related parties should consider the implementation of tele-technology in the advancement of the biopsychosocial model. Since a recent study found group CPAP education enhanced acceptance of therapy, future studies can investigate educational interventions with peer support through social app platforms (72). Integration of continuous tele-education and online motivational interviews might maximize the positive effect on patients’ health behavior by enhancing their confidence level about the therapy (65, 71). We also suggest the inclusion of a polysomnography chart in the educational video, as one RCT found that this increased the mean usage hours (SG = 4.2 ± 2.5 h/night, interventional group = 5.2 ± 2.1 h/night, *p* = 0.027) and the compliance (SG = 68.3%, interventional group = 86.5%, *p* = 0.021) (73).

### Future of Telehealth Systems for Enhancing Continuous Positive Airway Pressure Adherence

**FIGURE 1:**
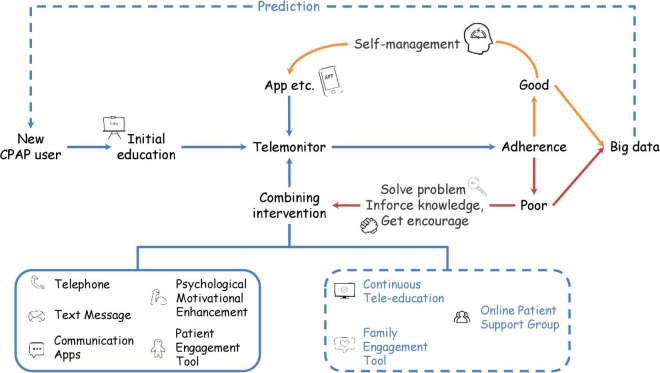
Flowchart for future telehealth application. New CPAP users receive education and initiate a self-management approach initially. Then, healthcare providers remotely monitor CPAP usage data and identify the patient’s adherence occasionally. For the patient with good adherence, healthcare providers administrate an app for the patient to self-manage. Patients with insufficient adherence should join combining interventions. Currently available interventions include telephone, text message, psychological motivational enhancement, and patient engagement tools. Future studies on continuous tele-education, family engagement tools, and online patient support groups are required. Collection of adherence data able to build big data. Training artificial intelligence with big data might help to predict adherence of new CPAP users. Blue words and dotted lines represent future interventions.

As individual treatments show heterogeneous results, we would like to propose an individualized telehealth model integrating all the interventions shown in Figure 1. Firstly, a new CPAP user should receive educational material in the form of a video, slideshow, or booklet. Then, adherence data should be transmitted to the cloud database, enabling HPs to analyze CPAP adherence. Patients with good compliance will use the apps for self-management and contact HPs when necessary. If HPs review the usage data periodically, then patients with poor adherence could receive the advice and combinations of interventions. Currently available interventions to combine included text message, telephone, motivational enhancement, and patient engagement tools. Developers and researchers could consider including family engagement tools (74), online patient support groups using telecommunication apps, and continuous tele-education in future research.

Moreover, the collection of adherence data from CPAP devices into a database also facilitates big data development (35, 38, 40, 47–51). The application of cloud based-data allows an overview of real-world data and investigation of CPAP adherence patterns (75, 76). Besides, big data shows the effectiveness of different interventions with minimal effort required, compared to the traditional observational studies (33). An analysis of AirView alone compares adherence patterns among various countries (*N* = 4,181,490) and supports the positive effects of a patient engagement tool (77). Big data from German homecare providers also reveals several predictors of poor CPAP adherence and investigated the effect of shifting therapy (78). The current benefits of big data analysis imply the worthiness of building more cloud databases and collecting various types of data.

**FIGURE 2:**
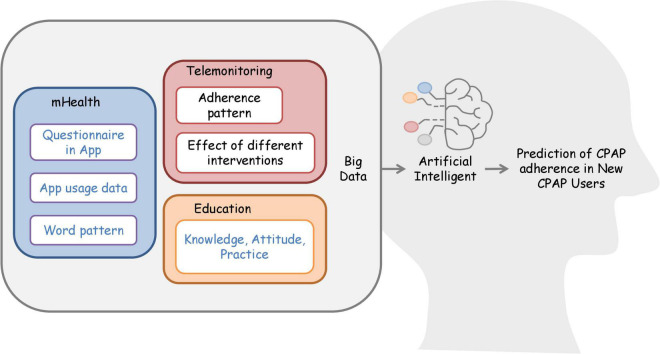
Integration of big data and artificial intelligence for the development of the predictive model. Collection of adherence data from telemonitoring able to generate adherence patterns database. Future studies can use the database to continue work on the artificial intelligence model to predict CPAP adherence. Researchers can also consider building biopsychosocial databases by retrieving data such as psychological, social, and clinical questionnaires in-app. Developers can collect app usage data and word patterns used in telecommunication apps to train the artificial intelligence model to predict CPAP adherence. For education, researchers could work on a prediction model based on the knowledge, attitude, and practice of CPAP users. The blue words represent future intervention.

Recently, some studies have developed artificial intelligence models to predict adherence (79–81). A model predicting the next-30-day adherence phenotype achieved the highest sensitivity (90%), specificity (96%), and accuracy (95%) (79). However, the model for 6-month adherence had a sensitivity ranging between 71 and 77% and a specificity ranging between 69 and 72% (80). These promising results encourage future studies to use big data to develop clinical prediction models to identify patients with likely poor adherence. Additionally, future research could collect various types of data to integrate the biopsychosocial model into clinical prediction models. For example, responses from clinical and psychological questionnaires, the app usage data, and word patterns of patients’ responses can build a database to train more artificial intelligence models. Investigation of knowledge, attitude, and practice of CPAP adherence also assists in the generation of these prediction models (Figure 2).

## Conclusion

Telemonitoring has been proven to improve the compliance of CPAP, resulting in the reduction of disease severity and side effects, and enhancement of social and psychological support. It also improves the quality of life and slightly alleviates the economic burden. We support the application of telemonitoring in CPAP management and following up. As tele-education alone is insufficient in improving CPAP adherence. Thus, we call for a bio-psycho-social care model integrating multiple interventions to promote better care for CPAP therapy.

## Author Contributions

BT, GL, JL, and QL: conception and design of the work. BT, GL, JL, CL, ST, HL, and QL: drafting of the manuscript and final approval for publication. BT, GL, JL, HL, and QL: critically revision for the content. All authors contributed to the article and approved the submitted version.

## Conflict of Interest

The authors declare that the research was conducted in the absence of any commercial or financial relationships that could be construed as a potential conflict of interest.

## Publisher’s Note

All claims expressed in this article are solely those of the authors and do not necessarily represent those of their affiliated organizations, or those of the publisher, the editors and the reviewers. Any product that may be evaluated in this article, or claim that may be made by its manufacturer, is not guaranteed or endorsed by the publisher.
